# Prevalence and associated factors of overweight and obesity among medical students from the Western Balkans (South-East Europe Region)

**DOI:** 10.1186/s12889-023-17389-7

**Published:** 2024-01-02

**Authors:** Miloš Ilić, Huiwen Pang, Tomislav Vlaški, Maja Grujičić, Budimka Novaković

**Affiliations:** 1 Department of Pharmacy, Faculty of Medicine, University of Novi Sad, Hajduk Veljkova 3, Novi Sad, 21000 Serbia; 2 Australian Institute for Bioengineering and Nanotechnology, The University of Queensland, Brisbane, QLD 4072 Australia; 3 School of Biomedical Sciences, Faculty of Medicine, The University of Queensland, Brisbane, QLD 4072 Australia; 4 Medical Faculty Heidelberg, University of Heidelberg, Im Neuenheimer Feld 672, 69120 Heidelberg, Germany; 5 Department of General Education Subjects, Faculty of Medicine, University of Novi Sad, Hajduk Veljkova 3, Novi Sad, 21000 Serbia

**Keywords:** Overweight and obesity, Medical students, Young adults, Public health, Western Balkans, South-East Europe

## Abstract

Being overweight and obese is associated with an elevated risk of developing noncommunicable diseases, which are the leading causes of mortality worldwide. It is a warning that global prevalence of overweight among university students ranges from 20 to 40%, which presents a significant public health problem. To date, there was no research conducted on medical students regarding the prevalence and associated factors of overweight and obesity in the countries of the Western Balkans (Slovenia, Croatia, Bosnia and Herzegovina, North Macedonia, and Serbia). The aim of this study was to determine the prevalence and potential demographic, socioeconomic, and health-related behavioral factors associated with overweight and obesity of medical students from Western Balkans. A cross-sectional study was conducted, surveying 2452 students from 14 medical faculties from 5 countries in the region. The prevalence of overweight was 12% and obesity was 2.3% among medical students from Western Balkans. Male gender and smoking status are significant positive predictors of overweight and obesity. Daily level of physical activity up to 1 h per day, going to preventive check-ups once a year or as a part of annual dormitory check-ups are associated with lower odds of being overweight and obese. By creating adequate public health educational programs, students can be influenced to acquire proper health-related lifestyle habits, which would lead to reducing the prevalence of overweight and obesity among the student population, as well as risk of developing noncommunicable diseases and improving the overall health of the population.

**Supplementary Information:**

The online version contains supplementary material available at 10.1186/s12889-023-17389-7.

## Background

The World Health Organization (WHO) defines overweight as a condition manifested by excessive adiposity and obesity as a chronic complex disease impairing health, characterized by further excessive adiposity [1]. The imbalance between energy input and expenditure (energy surplus) leads to fat deposits forming and results in overweight and obesity (OWOB) that can be caused by multiple factors, including genetics, obesogenic environments, and various psycho-social factors [2].

Being OWOB is associated with an elevated risk of developing non-communicable diseases (NCDs), including hypertension and cardiovascular diseases, dyslipidemia, insulin resistance, diabetes, neurological diseases such as Alzheimer’s, and other neurodegenerative diseases, osteoarthritis and other musculoskeletal problems, as well as malignant diseases, which are the leading causes of mortality worldwide [3, 4].

Typically, university students alter their lifestyle habits upon starting university education [5]. Students frequently consume high-energy diets rich in salt, followed by irregular meals, which, along with a lack of physical activity (PA), can have a negative effect on their health [6, 7].

Globally the prevalence of overweight among university students ranges from 20 to 40%, which presents a significant public health problem [8, 9]. Low PA levels, better socioeconomic status, rural origin, alcohol, tobacco, and drug abuse, as well as negative social impacts such as family, peers, and social media, are associated with OWOB among young people [5–12].

Research shows that the prevalence of harmful use of alcohol was 32.9%, and that smoking among medical students was present in 20%, with a significant increase in alcohol abuse and smoking in the student population over the past few years [13–18]. Students often misuse alcohol and smoking as dysfunctional and unhealthy ways to overcome stress caused by increased demands of studies, unsatisfactory quality of life, or to improve mood or reduce anxiety and that represents health risk behavior that is significantly associated with the higher risk of OWOB and subsequent development of NCDs [13–18].

According to the latest data from WHO European Regional Obesity Report 2022, 59% of adults (63% male, 54% female) in the European Region are OWOB. Age-standardized prevalence of OWOB among adults in 2016 was also severe in the countries of the Western Balkans region (Table 1) [19]. Globally, an estimated 5.02 million deaths were attributable to obesity in 2019 [20].

**Table 1:** Age-standardized prevalence of overweight and obesity among adults in countries of Western Balkan region, 2016 [16]

Country	Age-standardized prevalence (%)					
	Overweight (including obesity)			Obesity		
	Both sexes	Males	Females	Both sexes	Males	Females
Republic of Slovenia	56.1	62.1	49.9	20.2	19.4	21.0
Republic of Croatia	59.6	66.2	53.0	24.4	24.1	24.5
Bosnia and Herzegovina	53.3	59.7	47.0	17.9	17.1	18.4
Republic of North Macedonia	58.1	64.9	51.2	22.4	22.6	22.1
Republic of Serbia	57.1	63.8	50.5	21.5	21.1	21.8

Medical students, as tomorrow’s health professionals, are considered the most informed and health-conscious group [21]. Medical practitioners and students who adopt healthy lifestyle habits are more likely to feel confident when advising their patients on health behaviors, such as PA and diet. Additionally, patients are more likely to trust and follow the guidance provided by physicians who lead a healthy lifestyle [21, 22]. Health promotion programs in graduate medical schools should be prioritized as they will likely influence the patients population to adopt healthier behaviors [21].

However, to date, there was no research conducted on medical students regarding the prevalence and associated factors of OWOB in the countries of the Western Balkans (Republic of Slovenia, Republic of Croatia, Bosnia and Herzegovina, Republic of North Macedonia and Republic of Serbia).

The aim of this study was to determine the prevalence and potential demographic, socioeconomic, and health-related behavioral factors associated with OWOB of medical students from Western Balkans.

## Materials and methods

### Study design and population

Between November 2019 and February 2020, an observational, cross-sectional study was conducted with 2452 students from 14 medical faculties situated in the Western Balkans region:

- Republic of Slovenia:Faculty of Medicine at the University of Ljubljana;
- Republic of Croatia: Faculty of Pharmacy and Biochemistry at the University of Zagreb, Faculty of Medicine at the University of Rijeka;
- Bosnia and Herzegovina: Faculty of Medicine and Faculty of Pharmacy at the University of Sarajevo, Faculty of Health Studies at the University of Sarajevo, Faculty of Medicine at the University of Zenica, Faculty of Pharmacy at the University of Mostar, the Faculty of Health Studies at the University of Mostar;
- Republic of North Macedonia. Faculty of Medicine and Faculty of Pharmacy at the University “St. Cyril and Methodius” Skopje;
- Republic of Serbia: Faculty of Pharmacy at the University of Belgrade, Faculty of Medicine Novi Sad at the University of Novi Sad, and Faculty of Pharmacy at the University of Business Academy Novi Sad.

Medical faculties were chosen using the convenience sampling method. Using the power analysis method, it was determined that the sample size provided a confidence interval of 95% and a margin of error of 5–8%.

### Study questionnaire

To collect data, an online survey was conducted using Google forms. The survey was easily accessible from any device and was distributed through various channels, including email, social networks such as Facebook, and faculty websites. The respondents confirmed their participation by filling out the survey and forwarding their answers. Participation was voluntary, and respondents were allowed to withdraw at any time. Only fully completed questionnaires were recorded in the database for analysis. Once the study was completed, the database was downloaded as a Microsoft Excel sheet. The research method adhered to Google’s privacy policy, ensuring the respondents remained anonymous.

### Variables

The survey was split into two sections. The initial section collected data on the participants’ general characteristics, such as the faculty they attended, their gender, year of study, body height and weight, household income, and the type of settlement they lived in prior to enrolling in the university. The second section of the survey focused on topics such as the average level of PA per day, alcohol consumption, the participant’s smoking status, frequency of preventive check-ups, and daily screen time. The self-report research paradigm was implemented in this study.

Body mass index was calculated using the formula:

$$\mathrm{BMI}=\;(\mathrm{body}\;\mathrm{weight}\;\mathrm{in}\;\mathrm{kilograms})/{(\mathrm{body}\;\mathrm{height}\;\mathrm{in}\;\mathrm{meters})}^2$$

Participants were classified by their nutritional status based on the WHO recommendation: for BMI < 18.50 kg/m^2^ (underweight), 18.50-24.99 kg/m^2^ (normal weight), 25.0-29.99 kg/m^2^ (overweight), and ≥ 30.0 kg/m^2^ (obesity).

Medical students were divided into two groups based on their year of study: 1-3-year students and 4-6-year students. Based on self-evaluated household income (in relation to the average household income in the student’s country), participants were divided into three groups: below average, average, and above average. The type of settlement in which students lived before starting their university education was classified into two categories based on number of citizens: rural and urban. The average daily time spent engaging in PA was categorized into one of four options: no regular PA, up to 30 min per day, up to 1 h per day, and more than 1 h per day. Reported alcohol consumption was determined by selecting one of five answers: no alcohol consumption, occasional consumption, only on weekends, several times a week, or daily consumption. Based on the answers about the number of cigarettes smoked per day, students were placed into two categories: smokers (those who smoked occasionally or any number of cigarettes per day) and non-smokers. Students were asked about the frequency of preventive check-ups and could choose one of the answers: annual dormitory check-up only, several times a year, only as a part of public health action (preventive health check-ups or screening programs for early detection of illnesses like breast cancer, colorectal cancer, and other serious conditions etc.), once a year and never. Daily screen time was determined by checking one of the following choices: less than 1 h, 1-2 h, 2-3 h, 3-4 h and more than 4 h.

### Statistical analysis

The statistical analysis involved displaying categorical variables as frequencies and percentages. The association between variables was assessed using the χ2 test, and Cramer’s V was used as a measure of association.

To examine the relationship between the dependent variable (overweight and/or obesity) and independent variables (gender, year of study, daily level of PA, alcohol consumption, household income, type of settlement, student smoking status, frequency of preventive check-ups, and daily screen time), binary logistic regression (BLR) was used.

Initially, univariate BLR was conducted to examine the association of each independent variable with the outcome variable (overweight and/or obesity), with the reference category being “presence of OWOB”. Variables that showed a statistically significant association with the outcome variable were then included in a multivariate BLR model, controlling for the possible influence of other independent variables.

As statistical software SPSS Statistics for Windows ver. 24 (IBM Corporation) was used. *P* value < 0.05 was considered statistically significant.

### Ethical aspects of the research

The study was conducted according to the guidelines of the Declaration of Helsinki. Ethics Committee of the Faculty of Medicine University of Novi Sad waived the need for ethical approval for the study since the research did not incorporate invasive methods and did not violate the privacy of the respondents. Informed consent was obtained from all subjects.

## Results

The sample structure comprised 2015 (82.2%) female and 437 (17.8%) male students. Taking medical faculties into consideration, gender structure differed statistically significant (χ^2^ = 50.032, *p* < 0.001, fi = 0.143), where females were in the frequency range of 73.3% (Faculty of Medicine of the University of Zenica, Bosnia and Herzegovina) to 91.1% (Faculty of Medicine of the University of Rijeka, Republic of Croatia) (not shown in tables).

Within the sample of medical students, the prevalence of overweight was recorded at 12%, and obesity at 2.3% (not shown in tables). There was a significant difference between the faculties in relation to students’ nutritional status (χ^2^ = 54.695, *p* = 0.049, fi = 0.086) (Table 2). The lowest prevalence of OWOB students was among students of Faculty of Health Studies of the University of Mostar (7.6%, 0.0%), while the highest percentage of overweight students was at of Faculty of Pharmacy of the University of Belgrade (16.9%) and obese at Faculty of Pharmacy of the University of Sarajevo (4.1%) and Faculty of Pharmacy and Biochemistry of the University of Zagreb (4.1%), compared to respondents from other faculties.

**Table 2:** Nutritional status of medical students (*n* = 2452) in relation to attended medical faculties from the Western Balkans

Country	Faculty	Nutritional status								p*
		Underweight		Normal weight		Overweight		Obese		
		*n*	%	*n*	%	*n*	%	*n*	%	
Republic of Slovenia	Faculty of Medicine of the University of Ljubljana	13	6.0	180	82.6	21	9.6	4	1.8	0.049
Republic of Croatia	Faculty of Pharmacy and Biochemistry of the University of Zagreb	20	7.4	207	76.4	33	12.2	11	4.1	
	Faculty of Medicine of the University of Rijeka	15	10.3	112	76.7	18	12.3	1	0.7	
Bosnia and Herzegovina	Faculty of Medicine of the University of Sarajevo	14	10.9	97	75.8	14	10.9	3	2.3	
	Faculty of Pharmacy of the University of Sarajevo	11	9.1	86	71.1	19	15.7	5	4.1	
	Faculty of Health Studies of the University of Sarajevo	16	7.7	168	80.8	19	9.1	5	2.4	
	Faculty of Medicine of the University of Zenica	11	5.4	171	83.4	18	8.8	5	2.4	
	Faculty of Pharmacy of the University of Mostar	3	2.7	87	79.1	18	16.4	2	1.8	
	Faculty of Health Studies of the University of Mostar	11	10.4	87	82.1	8	7.6	0	0.0	
Republic of North Macedonia	Faculty of Medicine of the University “St. Cyril and Methodius” Skopje	14	7.8	131	73.2	27	15.1	7	3.9	
	Faculty of Pharmacy of the University “St. Cyril and Methodius” Skopje	11	8.7	98	77.2	17	13.4	1	0.8	
Republic of Serbia	Faculty of Pharmacy of the University of Belgrade	5	4.0	94	75.8	21	16.9	4	3.2	
	Faculty of Medicine Novi Sad of the University of Novi Sad	25	6.8	298	80.5	41	11.1	6	1.6	
	Faculty of Pharmacy of the University of Business Academy Novi Sad	1	0.7	114	82.0	21	15.1	3	2.2	

*p value calculated by using χ^2^ test for categorical variables. Significant at *p*<0.05

Male students were significantly more often overweight and obese compared to female students (25.6%/9.1%; 4.3%/1.9%) (χ^2^ = 120.05, *p* < 0.001, fi = 0.221) (Table 3). There was no statistically significant difference in nutritional status in relation to the year of study (χ^2^ = 3.552, *p* = 0.318, fi = 0.038), daily level of PA (χ^2^ = 14.153, *p* = 0.117, fi = 0.044), alcohol consumption (χ^2^ = 11.886, *p* = 0.455, fi = 0.040), household income (χ^2^ = 8.231, *p* = 0.222, fi = 0.041), type of settlement (χ^2^ = 4.235, *p* = 0.237, fi = 0.042), smoking status (χ^2^ = 7.601, *p* = 0.055, fi = 0.056), frequency of preventative check-ups (χ^2^ = 16.121, *p* = 0.186, fi = 0.047) and daily screen time (χ^2^ = 20.872, *p* = 0.052, fi = 0.053).

**Table 3:** Nutritional status of medical students (*n* = 2452) from the Western Balkans according to gender, year of study, daily level of physical activity, alcohol consumption, household income, type of settlement, smoking status, frequency of preventive check-ups and daily screen time

	Variables	Nutritional status								p*
		Underweight		Normal weight		Overweight		Obese		
		*n*	%	*n*	%	*n*	%	*n*	%	
Gender	Female	163	8.1	1631	80.9	183	9.1	38	1.9	< 0.001
	Male	7	1.6	299	68.4	112	25.6	19	4.3	
Year of study	1–3	110	6.7	1292	78.9	192	11.7	44	2.7	0.318
	4–6	60	7.4	638	78.4	103	12.7	13	1.6	
Daily level of physical activity	No regular physical activity	73	7.9	703	76.0	118	12.8	31	3.4	0.117
	Up to 30 min a day	28	5.8	383	79.1	64	13.2	9	1.9	
	Up to 1 h a day	36	7.1	411	81.2	53	10.5	6	1.2	
	More than 1 h a day	33	6.1	433	80.6	60	11.2	11	2.0	
Alcohol consumption	No alcohol consumption	59	7.2	644	78.3	101	12.3	18	2.2	0.455
	Occasionally	77	7.0	855	77.7	144	13.1	24	2.2	
	Only on weekends	25	5.8	352	81.3	41	9.5	15	3.5	
	Several times per week	8	9.4	70	82.4	7	8.2	0	0.0	
	Daily	1	8.3	9	75.0	2	16.7	0	0.0	
Household income	Below average	23	8.6	198	73.6	39	14.5	9	3.3	0.222
	Average	101	6.9	1169	79.6	162	11.0	36	2.5	
	Above average	46	6.4	563	78.7	94	13.1	12	1.7	
Type of settlement	Rural	61	8.1	586	77.6	86	11.4	22	2.9	0.237
	Urban	109	6.4	1344	79.2	209	12.3	35	2.1	
Smoking status	Non-smoker	139	7.3	1515	79.3	218	11.4	39	2.0	0.055
	Smoker	31	5.7	415	76.7	77	14.2	18	3.3	
Frequency of preventive check-ups	Never	14	6.1	170	74.2	37	16.2	8	3.5	0.186
	Annual dormitory check-ups only	83	7.0	941	79.3	140	11.8	22	1.9	
	Several times a year	20	6.8	231	78.3	31	10.5	13	4.4	
	Only as a part of public health action	3	6.4	34	72.3	9	19.1	1	2.1	
	Once a year	50	7.2	554	79.7	78	11.2	13	1.9	
Daily screen time	Less than 1 h	7	14.6	37	77.1	4	8.3	0	0.0	0.052
	1-2 h	17	5.5	247	79.9	39	12.6	6	1.9	
	2-3 h	54	8.5	487	76.7	82	12.9	12	1.9	
	3-4 h	58	6.6	705	80.7	95	10.9	16	1.8	
	More than 4 h	34	5.8	454	77.5	75	12.8	23	3.9	

*p value calculated by using χ^2^ test for categorical variables. Significant at *p*<0.05

 The results of univariate BLR analysis showed that gender, daily level of PA, smoking status, and frequency of preventive check-ups were significant predictors of OWOB (Table 4). The odds of being OWOB for male students were 3.475 times higher than for female students (95% CI:2.712–4.452; *p* < 0.001) as well as 1.371 times higher for smokers than non-smokers (95% CI: 1.060–1.774; *p* = 0.016). Students who had daily PA up to 1 h per day (OR:0.687, 95% CI:0.498–0.950, *p* = 0.023) and went to preventive check-ups once a year (OR:0.616, 95% CI:0.416–0.913, *p* = 0.016) or as a part of dormitory check-ups (OR:0.647, 95% CI: 0.449–0.932, *p* = 0.020) had lower odds of being OWOB.

In the multivariate BLR model, gender, daily level of PA and frequency of preventive check-ups remained as predictors of OWOB, while student smoking status was not a significant predictor. The odds of being OWOB for male students were 3.549 times higher than for female students (95% CI:2.872–4.832; *p* < 0.001). Students who had daily PA up to 1 h per day (OR:0.658, 95% CI:0.471–0.919, *p* = 0.014) and went to preventive check-ups as a part of dormitory check-ups (OR:0.637, 95% CI:0.435–0.933, *p* = 0.020) had lower odds of being OWOB.

Year of study, alcohol consumption, household income, type of settlement, and daily screen time were not significant predictors of OWOB.

**Table 4:** Association between independent variables and presence of overweight and obesity of medical students from the Western Balkans

Variables	Univariate Binary Logistic Regression				Multivariate Binary Logistic Regression			
	OR	95% CI for OR		*p*	OR	95% CI for OR		*p*
**Gender** (female vs. male)	3.475	2.712–4.452		< 0.001	3.549	2.872–4.832		< 0.001
**Year of study** (1–3 vs. 4–6)	0.987	0.777–1.255		0.917	1.046	0.812–1.338		0.720
**Daily level of physical activity**								
Up to 30 min per day	0.925	0.682–1.254		0.616	0.873	0.635–1.201		0.403
Up to 1 h per day	0.687	0.498–0.950		0.023	0.658	0.471–0.919		0.014
More than 1 h per day	0.794	0.585–1.077		0.137	0.674	0.489–0.927		0.927
No regular physical activity	1.00	.	.	.	1.00	.	.	.
**Alcohol consumption**								
Occasionally	1.065	0.826–1.373		0.628	1.054	0.808–1.374		0.699
Only on weekends	0.878	0.624–1.235		0.453	0.804	0.564–1.146		0.228
Several times a week	0.530	0.239–1.177		0.119	0.404	0.178–0.921		0.071
Daily	1.182	0.256–5.460		0.831	0.913	0.182–4.579		0.912
No alcohol consumption	1.00	.	.	.	1.00	.	.	.
**Household income**								
Average	0.718	0.508–1.015		0.061	0.705	0.493–1.009		0.056
Above average	0.801	0.515–1.165		0.246	0.743	0.503–1.099		0.137
Below average	1.00				1.00			
**Type of settlement (rural vs. urban)**	1.006	0.788–1.285		0.962	0.909	0.703–1.176		0.476
**Smoking status (non-smoker vs. smoker)**	1.371	1.060–1.774		0.016	1.221	0.932–1.598		0.147
**Frequency of preventive check-ups**								
Annual dormitory check-ups only	0.647	0.449–0.932		0.020	0.637	0.435–0.933		0.020
Several times a year	0.717	0.454–1.132		0.153	0.860	0.534–1.384		0.534
Only as a part of public health action	1.105	0.511–2.389		0.799	1.059	0.473–2.371		0.890
Once a year	0.616	0.416–0.913		0.016	0.708	0.470–1.068		0.099
Never	1.00	.	.	.	1.00	.	.	.
**Daily screen time**								
1-2 h	1.875	0.642–5.473		0.250	1.929	0.644–5.779		0.240
2-3 h	1.919	0.671–5.444		0.225	2.083	0.714–6.074		0.179
3-4 h	1.6	0.564–4.540		0.377	1.635	0.563–4.749		0.366
more than 4 h	2.209	0.776–6.289		0.138	2.183	0.749–6.359		0.152
Less than 1 h	1.00	.	.	.	1.00	.	.	.

*Abbreviations*: *OR *Odds ratio, *CI* Confidence interval

## Discussion

Our study shows that the percentage of normal weight and OWOB medical students are in accordance with the global average. The study by Pavičić-Žeželj et al. [23], conducted among students of the Faculty of Medicine in Rijeka (Croatia), shows that consistent with our results, most of the students have the normal weight, followed by students who are overweight and obese. Similar is stated in a study by Savić et al. [24], which was conducted among medical students in Banja Luka (Bosnia and Herzegovina), confirming that prevalence in our study corresponds with the prevalence of OWOB among medical students obtained in studies in the region of Western Balkans.

The result of our research shows that male students were more often OWOB compared to female students. Similar results from a study by Mahomed et al. [25] conducted in South Africa suggest that male medical students may be less concerned about their BMI than female students under faculty obligations, which could lead to males being more prone to OWOB than female students. A study conducted in Poland by Kanikowska et al. [26], came to the same observation. Yahia et al. [27], in a study conducted on university students in the USA, also found an association between the male gender and higher rates of OWOB among university students in relation to the female gender, which points to the ubiquity of this observation. Several theories may explain this association. Female students are more under social pressure to maintain a particular body image, which could be one of the factors for these findings [28]. It should also be taken into consideration that BMI is a population parameter [29] and cannot discern between individuals’ muscle or fat mass [30]. Since men tend to have a higher muscle mass percentage due to physiological differences, this could also be one of the proposed assumptions.

No significant difference in BMI in relation to household income and type of settlement was observed in our study. A study by Viñuela et al. [31], conducted between nursing students from Spain also found no significant difference, pointing out that other factors play a more critical role in developing obesity, such as poor nutrition, poor sleep quality, low PA, smoking, and drinking alcohol. However, a study by Asghar et al. [32] conducted in India indicated that dorm and hostel-dwelling medical students have a higher incidence of OWOB. These students tend to eat instant, high-calorie meals and have irregular meal patterns, which was one of the possible reasons that explain obtained results [32].

The obtained results from our research indicate no statistically significant difference in BMI in relation to smoking status and alcohol consumption. Ilić et al. [12] in a study on cigarette smoking among medical students from the Western Balkan showed no statistically significant difference in BMI regarding smoking status. A study by Rabanales-Sotos et al. [33] shows that medical students who are smokers have higher BMI values, pointing out that bad nutritional habits as well as other negative health-related lifestyle habits such as smoking, are highly associated with OWOB.

However, according to the BLR results in our study, controlling for the possible influence of other independent variables, smoking status was significant positive predictor of OWOB, while daily PA up to 1 h and frequency of preventive check-ups were associated with lower odds of OWOB among medical students in both logistic regression models. A study focused on adolescents and young adults by Jacobs [34] conducted in USA, indicates a positive association between smoking and BMI, but points out that, due to the complexity of other risk factors correlated with BMI, it is troublesome to observe a clear effect. Rabanales-Sotos et al. [33] indicates a strong association between bad health-related lifestyle habits such as smoking and OWOB among medical students. Smoking is associated with an increased likelihood of adopting behaviors that favor weight gain, such as engaging in low levels of physical activity [35], maintaining an unhealthy diet [36], and higher amounts of alcohol consummation [37], which may explain the increased odds for OWOB.

High alcohol consumption is another bad health-related habit. Although our results in this study indicated that alcohol consumption was not a significant positive predictor of OWOB, some studies suggest a positive correlation between high alcohol consumption and increased BMI [38–40]. Ekpanyaskul et al. [41], in a study conducted in Thailand, indicates that medical students who consume alcohol more often have a higher chance of being OWOB. Recent studies have established that alcohol disrupts lipid metabolism and can lead to fatty liver degeneration, insulin deregulation, and increased adipose tissue deposition [42, 43]. Thus, excessive alcohol intake may contribute to weight gain through various mechanisms, such as increased caloric intake [44], alterations in metabolism [45], and the promotion of adipogenesis [46]. However, obesity is a multifactorial condition in which the consumption of alcohol represents just one of the contributing factors. There are many confounders that researchers need to be aware of when trying to make a general conclusion, such as type of alcohol beverages, gender, level of PA, sleep duration, nutritional pattern, and genetic variability. Abstention and moderation in alcohol drinking remain the official recommendations [43].

The inverse relationship between PA and BMI has been widely reported by much research [10, 23, 47, 48]. Catovic and Halilović [47] in study conducted in Bosnia, and Hemmingsson et al. [48] in a study conducted in Sweden, suggest that high BMI is significantly associated with higher PA levels in obese individuals. This is in accordance with the results in a study by Ilić et al. [11] where the main motive for regular PA was to reduce weight in students who were OWOB and the PA motivation is stronger among female medical students from the Western Balkans than it is among their male counterparts. Our results showed that daily exercise of about 1 h may significantly reduce the odds of being OWOB, which provides practical evidence that up to one-hour PA is the most beneficial exercise duration for prevention of OWOB. While our study did not delve into the specific types of PA, it is noteworthy that different forms of exercise may exert distinct influences on BMI or obesity [49]. For example, Cox et al. [50] found that in contrast to walking, swimming demonstrated more pronounced benefits in terms of short-term improvements in body weight, body fat distribution, and insulin levels. Over the longer term, swimming exhibited superior effects on body weight and lipid measures [50]. Thus, future investigations considering the nuances of various exercise modalities could provide valuable insights into understanding their differential impacts on body weight and composition.

According to our results, preventive check-ups can also be helpful in preventing OWOB. Through preventive screenings and regular monitoring of body weight, individuals can be made aware of any potential health risks related to obesity and take proactive steps to manage their weight and prevent the onset of obesity-related conditions. In addition, healthcare professionals can provide guidance and resources to promote healthy lifestyle behaviors, such as regular exercise and a balanced diet, which can help prevent obesity.

Results of our research show that year of study, household income, type of settlement, and daily screen time were not significant predictors of OWOB. A study by Gazibara et al. [51] conducted among university students in Serbia found no correlation between place of residence and BMI, similar to the results of our study. When it comes to observing the effect of screen time on general health, several studies found a strong relationship between anxiety and depressive symptoms [52], as well as poor sleep quality [53] in students. Results of a study by Yamamoto et al. [54] conducted in Japan indicates that students living alone have higher odds of being overweight than those living with their families, which corresponds with a higher chance of eating regular and homemade prepared meals.

Strengths of research is that it was carried out prior to the COVID-19 pandemic, so gathered data can be used for comparation with results from the future studies on the medical student population in the Western Balkans that would enable us to discern whether changes in prevalence of OWOB have occurred due to shifts in the student population’s lifestyle over the past two years. Furthermore, our study lays the groundwork for future research aimed at evaluating the influence of different promotion programs or university education across different Western Balkan countries on the prevalence of OWOB within the student population. The main significance of our research lies in the comprehensive scope of the study, which was conducted for the first time in the Western Balkans counties. It focused on identifying factors that impact the prevalence of OWOB among the culturally specific medical student population in this region. Given that medical students represent the future of health workers, they play a important role in health promotion, as they possess the capacity to initiate crucial and ongoing public health initiatives to enhance lifestyle habits among students studying health-related subjects. The contribution of this study implies the potential application of its results in promoting national or international public health activities, particularly for future health workers and the broader student population.

Our study has several limitations that need to be acknowledged. The study design was cross-sectional, which limits our ability to make causal inferences or track changes over time [55]. Secondly, the accuracy and reliability of self-reported data could not be verified, despite our efforts to ensure anonymity and confidentiality. Thirdly, the sample of medical faculties was selected using the convenience sampling method and cannot be considered representative of all Western Balkan medical students.

## Conclusion

Among medical students from the Western Balkans, male gender and students’ smoking status are significant positive predictors of OWOB. Daily PA up to 1 h per day, going to preventive check-ups once a year or as a part of annual dormitory check-ups are associated with lower odds of being OWOB. The results of this research can be used for creating adequate public health educational programs on national or international level, particularly on the student population in order to influence positive change in health-related lifestyle habits, which can lead to reducing the prevalence of OWOB among the student population, lowering the risk of developing NCDs and consequently improving the overall health of the population.

### Supplementary Information

**Additional file 1**. Questinaire

## Data Availability

The data presented in this study are available upon reasonable request from the corresponding author.
