# Supplementary material for: Prevalence and associated factors of overweight and obesity among medical students from the Western Balkans (South-East Europe Region)
Source: BMC Public Health. 2024 Jan 2;24:29. doi: 10.1186/s12889-023-17389-7 (PMC10763029; doi:10.1186/s12889-023-17389-7)
Supplement: Supplementary file 1 — Additional file 1. Questinaire [file 12889_2023_17389_MOESM1_ESM.pdf]

Examining the life habits and attitudes of medical students toward public health action

Dear all, this is a survey that examines the life habits of medical students, the personal motivation of students to apply healthy lifestyle habits and the impact of external factors on the implementation of healthy lifestyle habits. The attitude of medical science students on the impact and effectiveness of existing public health actions on changing existing life habits is also examined.
The data obtained will serve to better set the goals of future public health actions and to improve the impact of public health promotions on the general population through the use of modern public health instruments.
The survey is anonymous and your participation is voluntary.
Estimated time to complete the survey is 7-13min.

* Означава обавезно питање

1. Select the faculty you are studying at: *

Означите само један овал.

- Medicinska fakulteta Univerza v Ljubljani
- Fakulteta za farmacijo Univerza v Ljubljani
- Fakulteta za zdravstvene vede Univerza v Mariboru
- Fakulteta za vede o zdravju Univerza na Primorskem
- Medicinski fakultet Univerziteta "Sv. Kiril I Metodij" Skoplje
- Stomatološki fakultet Univerziteta "Sv. Kiril I Metodij" Skoplje
- Farmaceutski fakultet Univerziteta "Sv. Kiril I Metodij" Skoplje
- Fakultet za medicinski nauki Univerzitet vo Tetovo

2. Choose your study direction: *

Означите само један овал.

- Pharmacy and related studies
- Medicine and related studies
- Dentistry and related studies
- Nursing and related studies
- Defectology and related studies

3. Year of study: *

Означите само један овал.

- 1
- 2
- 3
- 4
- 5
- 6

4. Gender: *

Означите само један овал.

- Male
- Female

5. Your height (cm): *

6. Your body weight (kg): *

7. Type of settlement where you lived before your studies: *

Означите само један овал.

- Village up to 500 inhabitants
- Village of 500 to 3,000 inhabitants
- Village over 3,000 inhabitants
- City up to 20,000 inhabitants
- City of 20,000 to 100,000 inhabitants
- City of 100,000 to 1 million inhabitants
- City of over 1 million inhabitants

8. Average household income: *

Означите само један овал.

- Far below average
- Below average
- Average
- Above average
- Far above average

Life habits

9. How many meals do you have during the day? *

Означите само један овал.

- 1
- 2
- 3
- 4
- 5
- More than 5

10. How often do you have breakfast? *

Означите само један овал.

- Every day
- 5-6 times a week
- 3-4 times a week
- 1-2 times a week
- I'm not having breakfast

11. How often do you consume raw fruits? *

Означите само један овал.

- Every day
- 3-4 times a week
- 1-2 times a week
- Less than once a week
- I do not consume raw fruits

12. How often do you consume raw vegetables? *

Означите само један овал.

- Every day
- 3-4 times a week
- 1-2 times a week
- Less than once a week
- I do not consume raw vegetables

13. How often do you eat meat? *

Означите само један овал.

- Several times a day
- Each day for one meal
- Several times a week
- I don't eat meat

14. How often do you consume fish? *

Означите само један овал.

- Several times a week
- 1 per week
- Less than once a week
- I don't eat fish

15. How often do you consume fast food? *

Означите само један овал.

- Every day
- Almost every day
- Several times a week
- Occasionally
- I don’t eat fast food

16. How often do you consume sweets and / or snacks? *

Означите само један овал.

- Every day
- Several times a week
- Less than once a week
- I don’t eat sweets and / or snacks

17. How much fluid do you intake during the day? *

Означите само један овал.

- Less than 0.5l
- 0,5 - 1l
- 1-2l
- more than 2l

18. Of the total amount of liquid you drink during the day, how much tap water is there? *

Означите само један овал.

- Less than 0.5l
- 0,5-1l
- 1-2l
- More than 2l

19. How often do you consume non-alcoholic sweetened beverages? *

Означите само један овал.

- Every day
- Several times a week
- Occasionally
- Never

20. If you consume alcohol, how often do you consume it? *

Означите само један овал.

- Occasionally
- On weekends
- Several times a week
- Every day
- I do not consume alcohol

21. How much time on average do you spend daily in physical activity ? *

Означите само један овал.

- 3 or more hours per day
- 1-2h daily
- Up to 1h daily
- Up to 30min daily
- I have no regular physical activity

22. How much time does your screen time take? *

"Screen time" - time spent on the screen, that is, on the computer, mobile phone, TV ...

Означите само један овал.

- More than 5h
- 3-4h
- 2-3h
- 1-2h
- Less than 1h

23. If you are a smoker, how many cigarettes do you smoke a day? *

Означите само један овал.

- I'm not a smoker
- I smoke occasionally
- Up to 5 cigarettes a day
- 5-10 cigarettes a day
- 10-20 cigarettes a day
- More than 20 cigarettes a day

24. How often do you go to preventative checkups? *

Означите само један овал.

- Once a year
- Several times a year
- Only when there is actual public health action
- Only when I have to do a systematic examination because of college / student dorm / driving ...
- I've never been to a preventative checkup

Motivation

25. When I find out that a food item is a health risk, I ... *

Означите само један овал.

- I do not consume it anymore
- I still consume it
- I occasionally consume it

26. I believe foods that are marketed as healthy are healthy *

Означите само један овал.

	1	2	3	4	5	
I tot:	◯	◯	◯	◯	◯	I totally agree

27. I have regular physical activity because: *

You can mark multiple answers.

Изаберите све што важи.

- ☐ I want to look better
- ☐ I want to feel better
- ☐ It helps me to control chronic illness
- ☐ It helps me to reduce stress
- ☐ I want to lose weight
- ☐ I have no regular physical activity

28. I would love to quit smoking, but I need professional help. *

Означите само један овал.

- ◯ Yes
- ◯ No
- ◯ I don't smoke

29. If I smoke a cigarette occasionally, it will not affect my health. *

Означите само један овал.

- ◯ I agree
- ◯ I do not agree

30. When I do not have health problems, I do not think about a healthy lifestyle. *

Означите само један овал.

	1	2	3	4	5	
I tot:	◯	◯	◯	◯	◯	I totally agree

31. I believe that I will live long if I improve my life habits. *

Означите само један овал.

	1	2	3	4	5	
I tot:	◯	◯	◯	◯	◯	I totally agree

32. I believe that I will improve my quality of life if I improve my living habits. *

Означите само један овал.

	1	2	3	4	5	
I tot:	◯	◯	◯	◯	◯	I totally agree

33. Would you like to get healthy lifestyle advice? *

Означите само један овал.

- ◯ Yes
- ◯ No
- ◯ I belive I have a healthy lifestyle

34. I find that I am not persistent enough to adhere to healthy lifestyle habits. *

Означите само један овал.

	1	2	3	4	5	
I tot:	◯	◯	◯	◯	◯	I totally agree

External factors

35. What are the reasons for your malnutrition? *

You can mark multiple answers.

Изаберите све што важи.

- [ ] My family does not eat healthy
- [ ] In the student restaurant, I do not have the ability to eat healthy meals
- [ ] I have no time because of my commitments in college
- [ ] My society does not feed that way
- [ ] When I stick to proper nutrition, people think I'm weird
- [ ] I have no motivation to change my life habits
- [ ] I am satisfied with my current appearance and health and have no need to change my life habits
- [ ] I can't resist "junk food"
- [ ] I do not have money
- [ ] I manage to eat properly

36. What is the most important factor when choosing a meal? *

Означите само један овал.

- ◯ That the meal can satiate me enough
- ◯ Price
- ◯ Speed of preparation
- ◯ That it's something I like to eat
- ◯ That the meal contributes to good health
- ◯ That the meal is low in calories

37. With whome do you most often consume fast food? *

Означите само један овал.

- Alone
- With friends
- With family
- With boyfriend / girlfriend
- Second
- I don’t eat fast food

38. I have no regular physical activity due to: *

Изаберите све што важи.

- Obligations on faculty
- Life situation
- Financial condition
- Current health status
- Second
- I have regular physical activity

39. Are any of your parents a smoker? *

Означите само један овал.

- Mother
- Father
- Both
- None

40. If there was a ban on smoking in public places I would ... *

Означите само један овал.

- I quit smoking
- I would reduce smoking
- It would not significantly affect the number of cigarettes I smoke
- I'm not a smoker

41. I have gained a health attitude in my childhood and I will hardly change it. *

Означите само један овал.

	1	2	3	4	5	
I tot:						I totally agree

42. There is a lot of information out there on what is healthy / unhealthy so I don't know what to believe.

Означите само један овал.

	1	2	3	4	5	
I tot:						I totally agree

43. Social networks can have a positive impact on raising awareness of healthy lifestyle habits. *

Означите само један овал.

	1	2	3	4	5	
I tot:						I totally agree

44. I believe that a healthy lifestyle requires ... *

You can mark multiple answers.

Изаберите све што важи.

- [ ] Enough money
- [ ] Enough free time
- [ ] Higher education
- [ ] Good childhood education
- [ ] Adequate education in primary and secondary schools
- [ ] Going to preventive checkups
- [ ] Continuous education of the general population on health
- [ ] Effective public health actions

Attitudes towards public health actions

45. Can you remember the public health action you last encountered? *

Означите само један овал.

- Yes
- No

46. If yes to the previous question, please write the public health action you last encountered.

____

47. How much has that public health action affected you?

Означите само један овал.

	1	2	3	4	5	
It m[illegible]	○	○	○	○	○	It was very influential

48. Public health actions must adapt to the age in which we live and the generation to which they are intended. *

Означите само један овал.

	1	2	3	4	5	
I tot:	◯	◯	◯	◯	◯	I totally agree

49. Public health actions should be customized to the target population. *

Означите само један овал.

	1	2	3	4	5	
I tot:	◯	◯	◯	◯	◯	I totally agree

50. The success of public health action depends most on: *

Означите само један овал.

- ◯ Action visibility
- ◯ Creativity in how content is transmitted
- ◯ Choosing of the target group
- ◯ Formal education
- ◯ Non-formal education
- ◯ By passing a law

51. I will read health information if it "pops up" on social networks. *

Означите само један овал.

	1	2	3	4	5	
I tot:	◯	◯	◯	◯	◯	I totally agree

52. I would use a mobile app to help me acquire healthy lifestyle habits. *

Означите само један овал.

	1	2	3	4	5	
I tot:	◯	◯	◯	◯	◯	I totally agree

53. I believe that mandatory health education will have a greater effect on the general population than leaving a choice for free information. *

Означите само један овал.

	1	2	3	4	5	
I tot:	◯	◯	◯	◯	◯	I totally agree

54. Introducing Health Education in Primary and Secondary Schools would enhance knowledge about healthy lifestyles. *

Означите само један овал.

	1	2	3	4	5	
I tot:	◯	◯	◯	◯	◯	I totally agree

55. As part of formal education or later employment, workshops should be held to enhance the healthy lifestyle of students / employees. *

Означите само један овал.

	1	2	3	4	5	
I tot:	◯	◯	◯	◯	◯	I totally agree

56. Training of peer educators to educate the population about healthy lifestyle habits could improve public health. *

Означите само један овал.

	1	2	3	4	5	
I tot:	◯	◯	◯	◯	◯	I totally agree

57. Ineffective public health policy contributes to the many risks to health damage. *

Означите само један овал.

	1	2	3	4	5	
I tot:	◯	◯	◯	◯	◯	I totally agree

58. How can public health actions improve public health? Specify.

Google није направио овај садржај нити га одобрава.

Google упитници
